# Therapeutic Applications of Extracellular Vesicles for Myocardial Repair

**DOI:** 10.3389/fcvm.2021.758050

**Published:** 2021-12-09

**Authors:** Chunping Liu, Nathan Bayado, Dongyue He, Jie Li, Huiqi Chen, Longmei Li, Jinhua Li, Xinyao Long, Tingting Du, Jing Tang, Yue Dang, Zhijin Fan, Lei Wang, Phillip C. Yang

**Affiliations:** ^1^State Key Laboratory of Dampness Syndrome of Chinese Medicine, The Second Affiliated Hospital of Guangzhou University of Chinese Medicine, Guangzhou, China; ^2^State Key Laboratory of Quality Research in Chinese Medicine, Institute of Chinese Medical Sciences, University of Macau, Macau, China; ^3^Division of Cardiovascular Medicine, Department of Medicine, Stanford University School of Medicine, Stanford, CA, United States; ^4^School of Medicine, South China University of Technology, Guangzhou, China

**Keywords:** extracellular vesicles, myocardial repair, diagnosis and treatment, drug delivery system, engineering strategy

## Abstract

Cardiovascular disease is the leading cause of human death worldwide. Drug thrombolysis, percutaneous coronary intervention, coronary artery bypass grafting and other methods are used to restore blood perfusion for coronary artery stenosis and blockage. The treatments listed prolong lifespan, however, rate of mortality ultimately remains the same. This is due to the irreversible damage sustained by myocardium, in which millions of heart cells are lost during myocardial infarction. The lack of pragmatic methods of myocardial restoration remains the greatest challenge for effective treatment. Exosomes are small extracellular vesicles (EVs) actively secreted by all cell types that act as effective transmitters of biological signals which contribute to both reparative and pathological processes within the heart. Exosomes have become the focus of many researchers as a novel drug delivery system due to the advantages of low toxicity, little immunogenicity and good permeability. In this review, we discuss the progress and challenges of EVs in myocardial repair, and review the recent development of extracellular vesicle-loading systems based on their unique nanostructures and physiological functions, as well as the application of engineering modifications in the diagnosis and treatment of myocardial repair.

## Introduction

About 16.5 million people die of cardiovascular disease every year and is still the leading cause of death according to the Global Burden of Disease (GBD) study (1). The case fatality rate from cardiovascular diseases is expected to rise further due to unhealthy lifestyles and aging population. Ischemic heart disease can be divided into coronary artery disease and myocardial disease. Coronary artery occlusion results in blood flow restriction, leading to myocardial hypoxia and subsequent tissue death (2). The damage dealt to the ischemic myocardium becomes main contributor of deteriorating heart failure and eventual mortality. The adult human left ventricle contains about 2 to 4 billion cardiomyocytes which are terminally differentiated cells lacking the ability to re-enter the cell cycle and proliferate (3). Large numbers of cardiac muscle cells die when ischemia occurs and are eventually replaced with non-contractile scar tissue. Therefore, it is of utmost importance to explore novel and clinically pragmatic strategies for myocardial repair.

Extracellular vesicles (EVs) are a group of membranous vesicles released by all cell types. These vesicles can range in diameter from 30 to 1,000 nm (4, 5). Exosomes are a subset of extracellular vesicle about 30–150 nm in size, with characteristic transmembrane proteins, such as CD63. Microvesicles are another common EVs with a particle size of 100–1,000 nm. Different from exosomes, which are secreted by cells, microvesicles are formed by cell membrane bubbling. Due to the limitation of the separation method, generally obtained exosomes refer to a mixed population of small EVs (sEVs). Since most published data cannot accurately determine whether the function of exosomes is generic EV activity or exosome-specific activity, we thus chose here to use the generic term EVs to represent the types of vesicles isolated non-specifically. These can be isolated from amniotic fluid, urine, cerebrospinal fluid, lymph and other body fluids (6–9). As an important carrier of intercellular information exchange, EVs are widely involved in the processes of myocardial angiogenesis, myocardial fibrosis and immune inflammatory response, and are expected to become a new target for clinical treatment of cardiovascular diseases (10–13). EVs contain a large number of endogenous proteins with different functions, including: tetraspanins proteins, heat shock proteins, endogenous cellular proteins, and lipid-related proteins such as phospholipase. In addition, EVs contain a variety of different types of RNA molecules such as mRNAs, circRNAs, miRNAs, snoRNAs, lincRNAs, and rRNA (14–16). The biological effect of EVs is conferred upon delivery of these proteins and RNA molecules to the recipient cells. EVs are widely studied, can be targeted, biocompatible, and immunogenic, which provide a potential therapeutic tool for clinical cardiovascular diseases.

EVs play an important role in intercellular communication, providing a new acellular therapy (16–19). Although many advances have been made in basic research on EVs repairing damaged myocardium, there are still challenges in clinical use and some key questions remain to be answered. In view of the great potential of EVs for myocardial repair, with the continuous exploration and solution of key problems, it will have a very broad clinical prospect. In this review, we discuss the advances and challenges of EVs in cardiovascular studies based on the structure and physiological function, development, advantages, engineering modification and application of EVs drug loading systems in myocardial repair and diagnosis.

## Mechanism of EVS Repairing Damaged Myocardium

**Figure 1:**
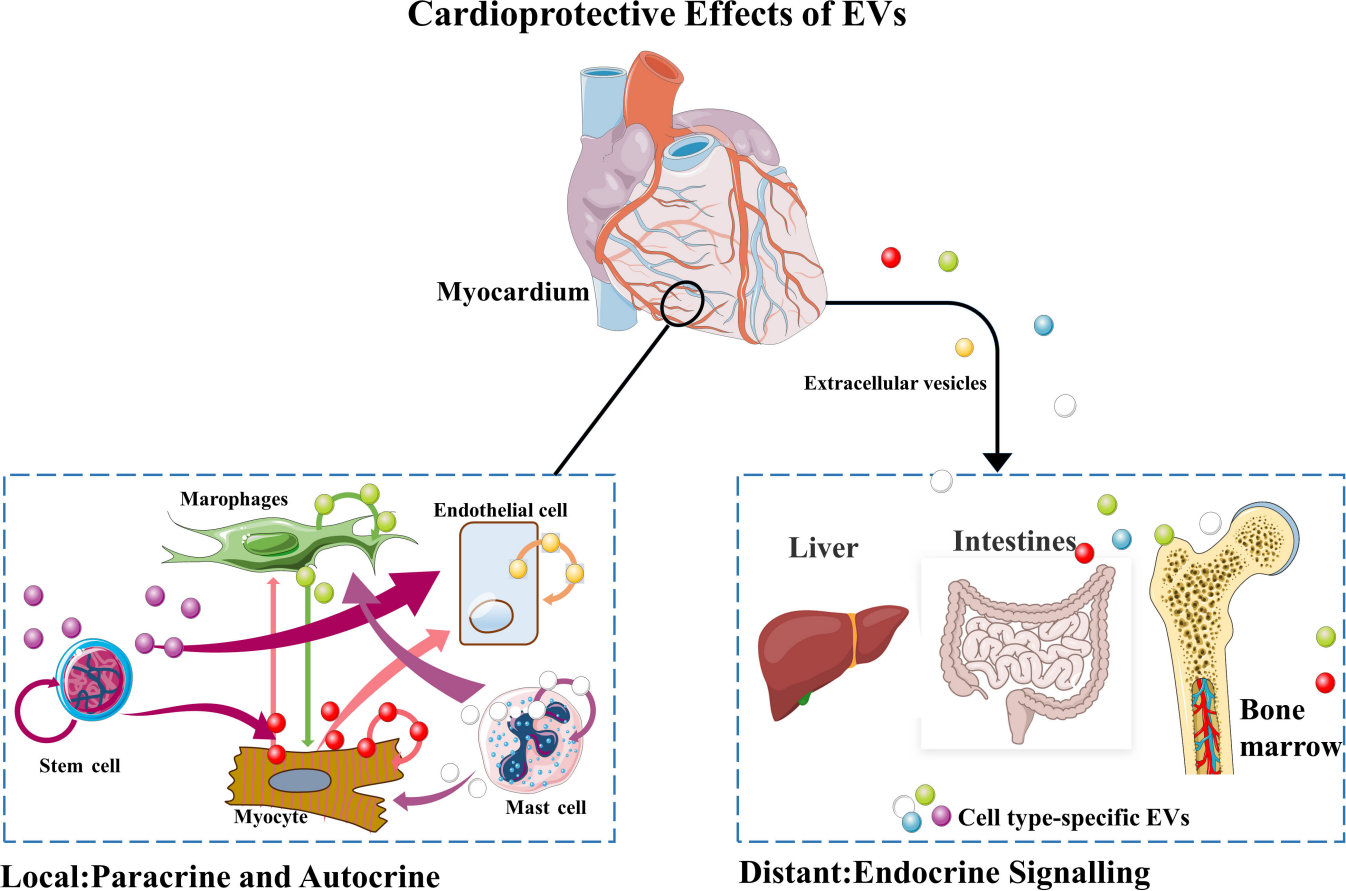
Mechanisms of EVs-mediated local and distal communications of the heart.

Since the discovery of EVs, basic research on EVs have emerged especially in the field of cardiovascular disease. The role of EVs in cardiovascular disease varies due to the diversity of their origin, production pathway and content. As shown in Figure 1, EVs that have therapeutic effects on ischemic myocardium include local source (for example, paracrine and autocrine) and distant source (for example, mesenchymal stem cells derived from bone marrow). Recent studies have found that some clinical drugs can play a cardioprotective role by regulating EVs production. For example, Lionetti has demonstrated that conventional cardiovascular drugs increase release of EVs promoting proliferation of cardiac progenitor cells (20). More interestingly, changes in the *in vivo* distribution of EVs may also play a role in cardiac protection (21). These studies further illustrate the important roles of EVs in myocardial repair. In this section, we will summarize the mechanisms of EVs of different origins in myocardial repair.

### Role of Cardiomyocyte-Derived EVs in Myocardial Repair

EVs are involved in the process of cardiovascular pathology, the communication between cardiomyocytes, fibroblasts, smooth muscle cells and endothelial cells, and the regulation of cardiac regeneration, ventricular remodeling and angiogenesis (22–24). Cardiomyocytes are the main cells of the heart and their EVs play different roles in myocardial repair (25). Myocardial ischemia and I/R injury are the main causes of myocardial injury. And in this part, we mainly describe the effect of cardiomyocyte-derived EVs on injured myocardium.

Myocardial infarction is a common manifestation of ischemic heart disease/coronary artery disease (26). It is characterized by sudden interruption of coronary blood flow and necrosis of supplying cardiomyocytes. Although primary coronary angioplasty and drug therapy repair the impaired cardiac function to some extent, the mortality rate remains high. More and more evidences have shown that paracrine factors play an important role in the process of myocardial infarction, the changes of microRNAs (miRs) in circulation can accurately reflect the myocardial injury *in vivo* (4), and the nearby living myocardium can protect myocardial cells from hypertrophy by capturing EVs. Circulating miRs released by the damaged myocardium after acute myocardial infarction (AMI) can also be transferred to distal organs through circulation via exosomes and affect the biological activity of recipient cells functionally. Yang et al. (27) found that miR-30 was highly enriched in exosomes isolated from serum of AMI patients or hypoxic cardiomyocyte culture medium, and miR-30a mediated up-regulation of core autophagy regulators Beclin-1, Atg12 and LC3II/LC3I. Regulating autophagy through exosomes is also a promising strategy for the treatment of ischemic heart disease. It has been found that rat cardiomyocytes can promote survival and inhibit apoptosis by releasing a variety of paracrine factors, including insulin-like growth factor-1, vascular endothelial growth factor (VEGF) and transforming growth factor-β, under co-culture conditions (28, 29). Cardiac Progenitor Cells participate in cardiac function recovery by secreting EVs (CPC EVs) through a paracrine mechanism. CPC EVs can reduce scar formation, alleviate undesirable remodeling, and improve cardiac function in acute and chronic myocardial infarction models (28). These findings are supported by other preclinical models of myocardial infarction (26).

The process of myocardial ischemia/reperfusion (I/R) involves injury of endothelial cell function and structure in a number of ways, including mass release of reactive oxygen species (ROS), direct injury of white blood cells, calcium overload, and reduced secretion of NO. EVs derived from cardiomyocytes were initially discovered under hypoxia and re-oxygenation conditions (6–8). Cardiomyocytes secreted exosomes containing circHIPK3 under hypoxic conditions to protect injured cardiomyocytes. In addition, the inflammatory response is involved in all aspects of I/R injury (25, 30). Chen et al. (31) found that eNOS activation in cardiac microvascular endothelial cells (CMECs) required a crosstalk between cardiomyocytes (CMs) and CMECs through the uptake of CM-derived sEVs. Tongxinluo induced CM-sEVs contain increased levels of long Intergenic non-protein coding RNA, regulator of reprogramming (Linc-ROR). Upon uptake into CMECs, linc-ROR downregulates its target miR-145-5p leading to activation of the eNOS pathway by facilitating the expression of p70s6k1 in these cells. The activation of CMEC-derived eNOS works to increase survival in both the CMECs and the CMs themselves.

### Role of Immune EVs in Myocardial Repair

Immune cells play an important regulatory role in the development of myocardial ischemia, I/R injury, septic cardiomyopathy and chemotherapy-related cardiomyopathy (32–34). EVs derived from immune cells show pleiotropism in pathological states. EVs have therapeutic potential of anti-apoptosis and anti-fibrosis, promoting angiogenesis, inhibiting ventricular remodeling, improving cardiac function and inhibiting local inflammatory response (35).

In the process of myocardial injury, macrophages are recruited to the damaged area to initiate release EVs into the peripheral tissue. Macrophage-derived EVs aggravate myocardial injury, inflammation, and promote myocardial fibrosis. Studies demonstrate that in the process of myocardial ischemia, macrophage derived exosomes deliver miR-155 to cardiac fibroblasts, which inhibit proliferation and promoted inflammation, suggesting that macrophage derived EVs containing miR-155 aggravate myocardial injury (36). Macrophages themselves are also receptors for miR-155, and endothelial cell derived EVs containing miR-155 can promote macrophage polarization (37).

Antigen presenting T cells have the ability to release specific EVs. Treg cells can improve the healing and remodeling after myocardial infarction (38, 39) and delays the progression of atherosclerosis (40). However, compared with cardiovascular diseases, Treg-derived exosomes are more recognized in organ transplantation and have a greater application prospect (41–44). The regulatory effect of DC-based EVs on the heart may depend on Treg activation, but it is still difficult to determine whether Treg exosomes have a cardioprotective effect. Studies have shown that Treg inhibits the effect of other T cells (43, 44), such as Th1 cells dependent on the transfer of exosomal miRNAs to receptor cells (45). Neutrophils are the most abundant white blood cells in human peripheral blood, accounting for about 50 to 70% of the total number of white blood cells. They play an important role in the innate immune system and are the first line of defense for the body to respond to the invasion of pathogens. They can resist the invasion of external pathogens through various ways such as phagocytosis, degranulation and production of reactive oxygen species (46). In the early stage of myocardial infarction, neutrophils and monocytes rapidly infiltrate the infarct region (47), releasing inflammatory EVs and triggering an inflammatory cascade (48). Neutrophil-derived and mast cell-derived EVs play an important role in initiating injury-related molecular patterns (DAMPs) of the innate immune response.

### Stem Cell-Derived EVs in Myocardial Repair

Different types of stem cell (SC) derived EVs can convey different biological information. EVs with cardioprotective function may come from marrow mesenchymal stem cell (MSC), embryonic stem cells, and hematopoietic stem cells. Various strategies have been tried for the treatment of cardiovascular disease with SC transplantation therapy. Studies have found that the paracrine factors of transplanted cells, not the transplanted cells themselves, play a major role in repairing damaged tissue.

#### Cardioprotective Effects of Embryonic Stem Cells (ESC), Induced Pluripotent Stem Cells (iPSC), and Their Derrivatives

Khan et al. (49) found that ESC exosomes enhanced angiogenesis, cardiac progenitor cells (CPC) survival, proliferation and cardiac repair after myocardial infarction, and also participated in anti-inflammatory effects, enhanced cardiac function and reduced fibrosis. Wang et al. reported that iPSC exosomes protected cardiomyocytes from H_2_O_2_-induced oxidative stress by inhibiting caspase 3/7 activation, and alleviated IR injury in mouse myocardium by delivering cardiac protective miRs such as miR-21 and miR-210 (50). iPSC-derived cardiomyocytes from human placental amniotic mesenchymal stem cells were successfully implanted in mice to improve myocardial activity and cardiac function after myocardial infarction (51). Transplantation of ESC and iPSC derived cardiomyocytes has been employed, however problems such as arrythmia and poor retention of transplanted cells over time limit practical clinical applications (52, 53). iPSC/ESC cardiomyocyte EV therapy improves heart function without the risk of poor engraftment and induction of arrythmia, while allowing for the generation of patient specific EVs.

#### Properties of Multipotent CPC and MSC Derived EVs

Studies on CPCs and their derived exosomes therapeutic potential have demonstrated improvements in cardiac function (54). Physoxic conditions (5% O_2_) in cultured CPCs increase the number of EVs released while maintaining basal cell gene expression and cell morphology as opposed to hypoxic conditions (55), demonstrating changes in the microenvironment of EV donor cells modulate dosage of EV release in specific contexts. Therefore, regulation of CPC secretion can affect the paracrine potential of their EVs.

MSC-EVs has been shown to have similar or even better therapeutic activity than parent MSCs in inhibiting inflammation, oxidative damage and the proliferation of fibrosis in damaged tissues (56, 57). In ischemic cardiovascular disease, MSC-EV therapy reduces cardiomyocyte apoptosis, thereby reducing the extent of infarction and improving functional recovery and new vessel formation. Bone marrow derived MSCs have immunosuppressive properties, and the use of MSC-derived EVs alone can avoid the occurrence of immune rejection and enhance the repair of damaged cardiac muscle (58). Ju et al. (59) demonstrate that intramyocardial injection of MSC-derived exosomes can promote myocardial cell proliferation and angiogenesis, while reducing infarct size in mice with myocardial infarction. These results suggest that MSC-derived exosomes can play a protective role in the early stage of myocardial infarction.

## Engineering Strategies of EVS in the Diagnosis And Treatment Of Cardiovascular Diseases

To improve the therapeutic effect of chemical and biomolecular drugs, researchers have used nanoparticles of various scales as drug carriers (60). However, the clinical transformation of vectors faces two major problems: Safety of materials and vectors and rapid clearance of reticuloendothelial system (RES) or mononuclear Phagocyte system (MPS) (61). Compared with nano-carriers constructed from artificial materials, endogenous nano-carriers have the advantage of biocompatibility *in vivo* and have broad prospects in improving drug delivery and therapeutic effect. EVs, as nanocarriers, have the advantages of being similar to cell membranes, small in size, negatively charge, less recognized by immune cells, and penetration of deep tissues (62). Therefore, EVs may serve as an ideal natural nanomaterial for the delivery of myocardial repair drugs (Table 1).

**Table 1:** Summary of application of extracellular vesicles (EVs) as carriers in myocardial repair.

**Origin**	**Isolation strategy**	**Type and size**	**Cargo loading**	**Type of disease**	**clinical outcomes**	**References**
Mesenchymal stem cell	Centrifugation Total Exosome Isolation reagent (Invitrogen)	Exosomes 135 nm	Lamp2b+IMTP transfection	AMI	IMTP-exosomes exert enhanced therapeutic effects	(63)
Induced pluripotent stem cell–derived cardiomyocytes (iCMs)	Differential ultracentrifugation method	EVs 98–677 nm	Mitochondrion iCMs self-contain	Myocardial infarction	M-EVs improve mitochondrial function and prevent post-MI LV remodeling	(64)
Mesenchymal stem cell	Ultracentrifugation	EVs		Chronic myocardial ischemia	Mesenchymal cell–derived EVs induct capillary and arteriolar growth resulting in increased cardiac output and stroke volume	(65)
Genetically modified MSCs overexpressing CD47	Ultracentrifugation	EVs 90–350 nm	miR-21 Electroporation	I/R injury	miR21-loaded CD47-Evs exert anti-apoptosis effects, alleviate cardiac inflammation, improve cardiac morphology and the functional recovery of the I/R myocardium	(66)
Mesenchymal stem cell Raw 264.7	Exosome isolation kit (Beyotime, China) LiposoFast extruder apparatus (Avestin, Canada)	Hybrid EVs 109.76 nm	RAW 264.7 membrane fusion-extrusion	I/R injury	Mon-Exos were shown to promote endothelial maturation during angiogenesis and modulate macrophage subpopulations after MI/RI offering additional techniques to help clinicians better manage regenerative therapeutics for ischemic heart diseases	(58)
	Ultrafiltration by centrifugation (UFC)	Chimeric EVs 30–150 nm	DPS/ischemic homing peptide/incubation	I/R injury	IschCDC-EVs greatly enhances localization to injured myocardium as a potential targeting carrier of CVD	(67)
HEK293 cells expressing CTP-tagged FLAG-LAMP2b	Sartorius 10-kDa (5 L) poly- ether sulfone membranes	Chimeric EVs <150 nm	siRAGE-loaded C-sEVs	Myocarditis	C-sEVs may be a useful drug delivery vehicle for the treatment of heart disease	(68)

### Myocardial Repair Strategies Using EVs as Carriers

**Figure 2:**
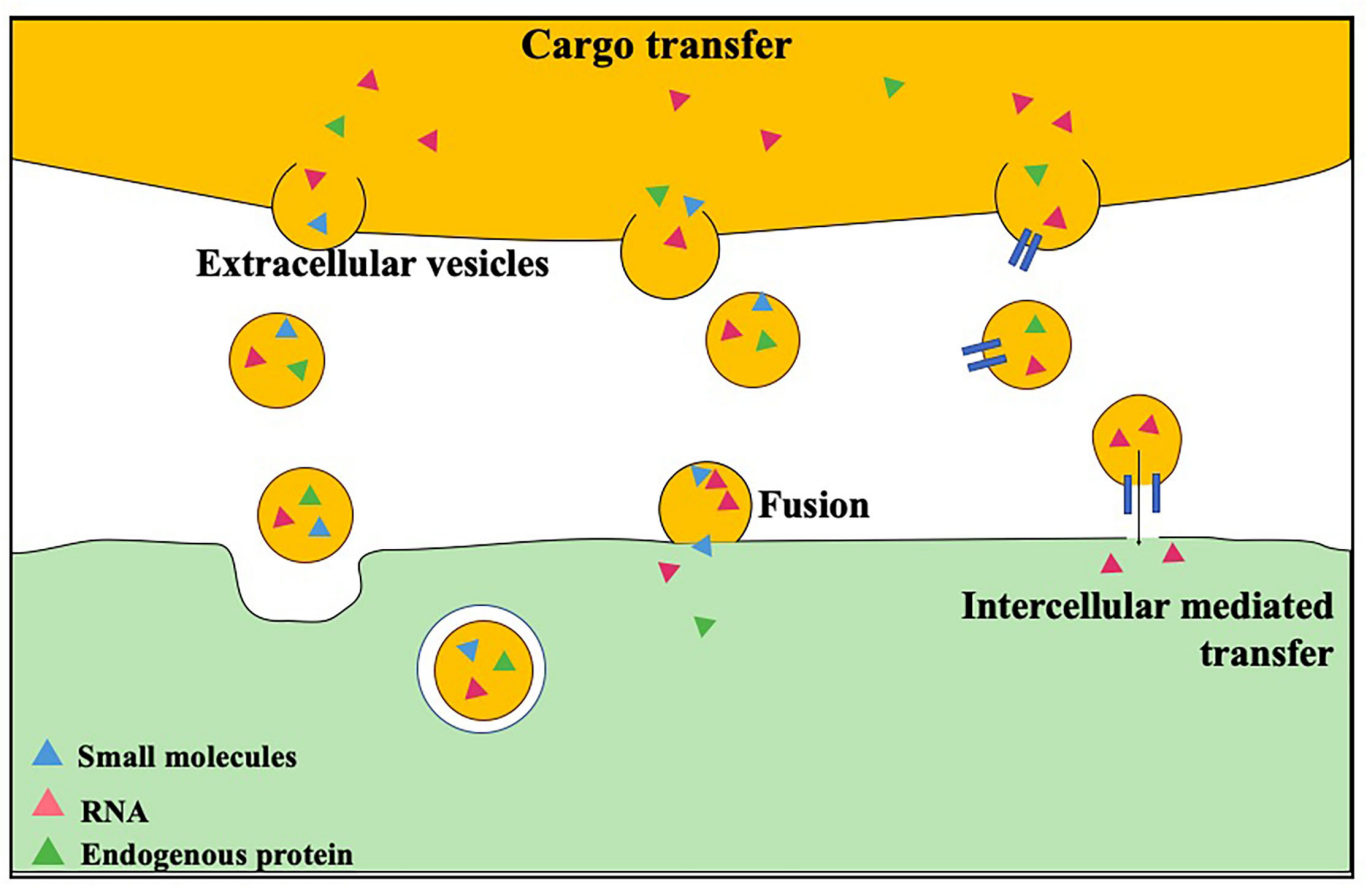
Loading and transshipment of EVs.

The research on EVs delivery of drugs is increasing gradually (69, 70). Some small molecule chemical drugs and gene drugs have been successfully loaded into EVs, showing great potential in the treatment of tumors, cardiovascular diseases and neurological diseases. EVs can accumulate drugs in treated cells, improve the stability and blood circulation time of small molecule drugs, and improve the efficacy of small molecule drugs (Figure 2). Sun et al. (71) showed that curcumin-loaded exosomes can increase the concentration of curcumin *in vivo*, increase the stability of the drug, and improve its anti-inflammatory and antioxidant effects.

RNA is an unstable macromolecular material making it difficult to achieve effective delivery. Existing deliveries include use of cationic liposomes, dendritic macromolecules, cationic polymer particles, but the carrier is still in the process of clinical application. This technology faces problems with security, stability, and tissue targeting (72). Overexpression of specific microRNAs via transfection in donor cells enables their packaging into EVs. For example, overexpression of miR-125b targeting SIRT7 in bone marrow MSC-derived exosomes down-regulated the levels of Bax, caspase-3 apoptotic proteins and IL-1β, IL-6, TNF-α inflammatory factors, and up-regulated the expression of Bcl-2, in order to repair the myocardial injury of I/R-rats (73). In addition, miR-181a delivered by exosomes of MSCs may inhibit the inflammatory response through c-Fos and promote the polarization of Treg cells to protect myocardial injury caused by miRs (74–77). These results suggest that donor cell engineering allows for directed cargo loading of small nucleotides into EVs, providing an effective therapeutic strategy for ischemia-reperfusion injury.

EVs can also be used as carriers of proteins for myocardial repair. Proteins can be genetically engineered from donor cells or encapsulated directly into EVs. In the former, transfected donor cells with a plasmid carrying the target gene are used to synthesize the protein encoded by the inserted gene. These proteins are loaded into exosomes. The supernatant of the cell culture is collected for isolation and purification of exosomes. In addition, proteins can be directly encapsulated in exosomes. Studies have shown that Yim et al. (78) encapsulated proteins in exosomes through an optically reversible protein-protein interaction, which significantly increased the level of target proteins in the receptor cells in *vivo* and in *vitro*, further proving the possibility of EV cargo design for not only nucleotides, but also protein.

### Design and Modification Strategies for Tissue Specific Targeting of EVs

EVs are considered as ideal natural drug carriers due to their good histocompatibility and low immunogenicity, but drug loading and targeted delivery are the problems that must be solved when EVs are used for cardiac repair. To complete drug loading and targeted delivery, researchers have paved the way for engineering EVs. For example, gene engineering technology can be used to express targeted proteins or peptides to the surface of EVs, which can improve the targeting effect of the drug and enhance the efficacy. However, the complexity of the genetic modification process has greatly hindered its widespread application. Recently, modification methods based on physical and chemical properties have been developed. In this section, we will summarize the design and modification strategies of EVs as therapeutic platforms. As shown in Table 2, we summarize strategies for the engineering of EVs.

**Table 2:** Strategies for cargo loading into EVs.

**Strategies^a^**	**Methods**	**Advantages**	**Disadvantages**	**Prominent examples**	**References**
Cargo loading into donor cells	Co-incubation	Simple and feasible; No damage to membrane integrity	Poor specificity; Low loading efficiency	Delivery of DHA and S1P	(79)
	Transfection	Simple and feasible; No damage to membrane integrity	Induce donor cell apoptosis; Impair biological responses; Inefficient packaging	Delivery of miRNA-181a, Lamp2b, IMTP and MiR21	(63, 74, 80)
Direct loading into EVs	Electroporation	Simple and quick; Higher loading efficiency than transfection	EVs aggregation; siRNA precipitation; Not suitable for some RNAs with special structures	Delivery of MiR21	(66)
	Extrusion	Efficient packaging	Cause potential damages to biofunctional contents	Targeted delivery of MSC-exosomes	(58)
	Freeze and thaw cycles	Higher loading efficiency	EVs aggregation; Lower drug loading capacity than extrusion	Delivery of curcumin and miR-144-3p	(81)
General modification of EVs membrane	Chimeric EVs	Cell membrane targeting ability	Cost of presenting chimeric peptides	Targeted delivery of MSCs and CDC-XOs	(82, 83)
New engineered EVs-based platforms	Hybrid EVs	Easy preparation and scalability; Adjustable physical parameters	May lose biological functions of integral EVs; Low homogeneity	Delivery of HELIOS	(84)
	New engineered EVs-based platforms EVs membrane camouflaged NVs	Maintain complex EVs membrane structure; Specific targeting ability; High therapy efficacy	Low scalability; Increase the difficulty of fabrication; Time-consuming	Delivery of MiR-21 mimics	(85)

a *CDC-XOs, cardiosphere-derived cell exosomes; CREKA, cysteine-arginine-glutamic acid-lysine-alanine; DHA, docosahexaenoic acid; HELIOS, highly efficient life-support intracellular opto-driven system; IMTP, ischemic myocardium-targeting peptide CSTSMLKAC; LFA1, lymphocyte function-associated antigen1 or αLβ2 integrin; Mac1, macrophage receptor 1 or integrin αMβ2; MSC, mesenchymal stem cell; NVs, nanovesicles; S1P, sphingosine-1 phosphate*.

Tian et al. (86) conjugated functional ligand RGDyk cyclic peptide to the surface of EVs through biological orthogonal reaction between the EVs with surface modification of cyclostyle and the azide polypeptide. They encapsulated them with curcumin to target the cerebral lesion area in a cerebral artery occlusion mouse model. This effectively inhibited inflammatory response and apoptosis in the focus region, and developed a novel exon-based targeted drug delivery vector for ischemic brain injury. Ligand fragments or homing peptides discovered by phage display and *in vivo* biopanning methods fused to the enriched molecules on the external part of exosomes have been exploited to improve the ability of exosomes to target specific tissues or organs carrying cognate receptors.

The homing peptide of 9AA (CSTSMLKAC, 9AA) can specifically target the myocardial cells of MIRI (87). Xu Wang used technology of molecular cloning and lentivirus packaging to engineer exosomal enriched membrane protein (Lamp2b) fused with ischemic myocardium-targeting peptide 9AA. The result found that exosomes engineered by IMTP can specially target ischemic myocardium, and mesenchymal stem cell-derived IMTP-exosomes exert enhanced therapeutic effects on AMI.

In addition to peptides, binding targeted proteins such as nanosomic and signal regulatory protein α to exosome surfaces has also become an effective targeting strategy (88). For cardiovascular diseases, gene modification can also be used to overexpress bioactive substances with cardio protective effects in exosomes, such as miRNA or proteins to enhance exosome mediated cardio protective effects and reverse the effect of the pathological environmental secretome. Ni et al. (89) found that tissue matrix metalloproteinase inhibitor 2 (TIMP2) modified MSCs derived exosomes from human umbilical cord blood activated the Akt/Sfrp2 pathway, inhibiting cardiomyocyte apoptosis and extracellular matrix remodeling while promoting angiogenesis, improving heart function following myocardial ischemia. Compared with normal HUC-EXO, HUC-ExotimP2 increased the number of CD31^+^ and lectin immune-active cells in myocardial infarction rats and promoted angiogenic activity in the infarct area.

Although exosomes have shown promising in the field of drug delivery, it is not easy to achieve specific targeting of exosomes through surface modification, and the reaction conditions need to be strictly controlled to avoid the destruction and aggregation of exosomes caused by improper temperature, pressure and osmotic pressure. At present, the efficiency of exosome targeted drug delivery is not ideal as modifications result in clearing by the immune system.

### Problems in the Preparation of EVs

EVs have favorable biocompatibility, non-immunogenicity and non-tumorigenesis. They are physiologically more stable than graphted cells, circulate throughout the body, and can cross the blood-brain barrier, and are more resistant to freezing and thawing than cells, have the advantage of long-term storage (90), and have natural properties for therapeutic use. In addition, EVs prove suitable for modification to deliver drugs to target cells (91). However, there is still a lack of standardized methods for the collection, separation and purification of EVs, and different separation methods lead to large differences in the purity, size and concentration of EVs (92), hindering the introduction of EVs into clinical practice.

The most common EVs separation technique is differential centrifugation, however, EVs obtained by this technique often contain aggregates of cell culture medium, cell proteins and particles (93). In addition, production of EVs is difficult to scale up due to time-consuming isolation process, low yield, and need for specialized equipment. Another common separation technique is based on monoclonal antibodies to isolate EVs-associated antigens (94). However, the disadvantage of this technique is low specificity: non-EVs materials or cells carrying the antigen can easily bind with the antibody, which greatly reduces the purity of the extracted EVs (95, 96). High performance liquid chromatography can provide highly purified EVs, but this technique also requires expensive equipment and low yields, which limits its widespread use (93). At present, there is still a lack of a consensus, standardized high specificity, high yield EVs isolation and purification method.

The lack of appropriate preservation methods is another major problem that limits the clinical use of EVs. Generally, EVs are stored at −80°C, become unstable under long term storage (97). Studies have shown that the surface and morphological characteristics of EVs change and the protein degrades after 4 days of storage at −80°C (98). The size of EVs decreased when stored at 4 or 37°C, indicating structural change or degradation. These studies show that the storage conditions of EVs are relatively harsh, which limits its clinical use (99).

Before clinical use of EVs in the treatment of cardiovascular diseases, there are still many difficulties to be solved: the criteria for isolation and identification, high yield and more economical protocols are still to be developed. Current storage conditions are harsh, and the *in vivo* dynamics of EVs have not been studied in detail. EVs have irreplaceable advantages in the treatment of cardiovascular diseases, including those diseases that lack effective drug therapy (100).

## Application of Engineered EVS in Myocardial Repair

Natural EVs are an ideal drug delivery system, but there are still some problems to be solved, such as separation, identification criteria, and high yield. The emergence of engineered EVs can overcome the challenges of mass production, identification criteria, and isolation and purification to advance the clinical application of exosomes. At the same time, the emergence of block copolymers provides the possibility for the personalized customization of engineered extracellular vesicle, improves the targeting of drugs, and enables the drugs to be specifically concentrated at the injured myocardium, thus increasing the intensity of action while reducing the dose of drugs.

### Preparation of Engineered EVs

Engineered EVs are nano-vesicles made of natural EVs or cell membranes by special methods. Engineered EVs can be divided into multilayer vesicles 50–1,000 nm and single-layer vesicles, in which single-layer vesicles are further divided into small vesicles (SUV) 20–100 nm, large vesicles (LUV) 100–1,000 nm and large vesicle (GUV) 1–200 μm (5, 101). Engineered EVs also need to be given targeted, stimulus-responsive properties. To target cardiomyocytes, EVs need to be modified by genetic engineering or chemistry.

The exosomal donor cells are genetically modified to express the targeted peptide, effectively expressing the targeted peptide on secreted exosomes (63). Kim et al. (102) used lentiviral vectors fused with lysosomal associated membrane glycoprotein 2B and ischemic myocardial targeting peptide to genetically modify bone marrow MSCs to express ischemic myocardial targeting peptide. Fluorescence microscopy imaging showed that the number of exosomes expressing ischemic myocardial targeting peptide was greater than that of natural exosomes. Li et al. (103) reported a programmed exosome that would provide human antigen R with extremely high affinity to RNA. HuR and exosome transmembrane protein CD9 sequence were fused to modify donor cells, and the modified donor cells expressed the fusion protein HuR-CD9. These generated exosomes actively increased RNA loading. Ohno et al. (104) transfected pDisplay vector with GE11 peptide specifically binding to expression of EGFR into HEK293 cells to successfully express G11 peptide on the isolated exosome membrane. In subsequent experiments, they demonstrated the inhibitory effect of EGF-specific exosomes delivered Let-7a miRNA to EGFR-expressing xenograft breast cancer tissue in RAG2 ^−/−^ mice. Gene modification in exosome donor cells can greatly improve the stability of functional exosomes, but it is expensive and time-consuming to perform gene manipulation in donor cells.

At present, there are two chemical modification methods for artificial vesicles: wet chemistry method and microfluidic technology (101). The wet chemical methods grouped with the thin-film hydration method is the most classic (105). This method can be used in the synthesis of multilayer vesicles (106). Hammons et al. (107) used the film hydration method to dissolve dioleyl-phosphatidylcholine and poly-oxyethylene in chloroform, which was dried by rotary evaporation and then vacuum drying. Next, aqueous solution containing carbon nanotube pore protein was used for hydration. Ultrasound was used to remove the film on the inner wall of the container and the sample was extruded through the 200 nm polycarbonate film with a micro extruder to prepare hybrid bionic vesicles (108). Based on the film hydration method, a team replaced the “skeleton” required for self-assembly with block copolymers instead of pure phospholipids which broadened the application scope of the original method (109). In 2018, inkjet printing technology began being used to prepare emulsions (110). Inkjet technology can print amphiphilic molecules directly onto the receiving medium to form vesicles (111), which can quickly encapsulate fragile biomolecules such as proteins or other biological complexes, maintaining some degree of structural integrity of these molecules (112).

### Engineered EVs for the Diagnosis and Treatment of Myocardial Repair

Currently, the reassembly of phospholipid membranes with different functions has become a new direction of engineered EVs, which makes it possible for EVs to have specificity, enabling repair and anti-inflammatory functions at the same time. However, there are few related studies, and they are mainly used in cardiac ischemia and ischemia reperfusion injury in cardiovascular diseases.

#### Use of Engineered EVs in the Repair of Injured Myocardium

MSC-derived exosomes have attracted attention as paracrine components that mediate cardiac stem cell repair (63, 113, 114). Zhang et al. (58) obtained monocyte cell membrane isolated from RAW264.7 monocyte macrophages and EVs derived from MSCs through the fusion and extrusion of 0.2 μm polycarbonate membrane to obtain a monocyte simulation MSC-EVs. And the analog can imitate monocyte recruitment characteristics, enhancing myocardial recovery after ischemia-reperfusion injury by increased targeting efficiency, reducing collagen volume, reversing left ventricular anterior wall hypertrophy, reducing inflammation reaction to protect heart function, and promoting the role of angiogenesis. It has previously been reported that MSC-derived exosomes can repair damaged tissues (56), but how to precisely deliver exosomes into recipient cells *in vivo* remains a problem. As early as 2002, Lestini et al. (113) showed that polypeptides can direct liposomes to receptors expressed on pathologically stimulated vascular cells. Based on this, in recent years, a peptide known as a homing peptide has been used in the treatment of myocardial ischemia. Wang et al. (63) fused an exosome enriched membrane protein (Lamp2b) with the ischemic myocardial homing peptide CSTSMLKAC (IMTP) using molecular cloning and lentiviral packaging techniques. The results showed that MSC-derived IMTP exosomes had enhanced therapeutic effect on AMI. Antes et al. (67) designed a modular extracellular vesicle-anchoring platform DPS composed of a combination of 1, 2-BIS (Dimethylphosphino)ethane, polyethylene glycol and Streptavidin. CDC-EVs loaded with ischemic myocardial homing peptide coupled with DPS have proven to enhance the localization of injured myocardium and play a better role in myocardial repair.

### Scaffolds for Controled Release and Local Targetting of EVs

To achieve precise drug delivery, engineered EVs need to add stimulus-response modules. Drug release can be achieved by temperature, light, ultrasound, magnetic field or electric field stimulation. This greatly improves the controllability of drug delivery, thus increasing drug targeting and reducing drug side effects to achieve more accurate and controllable treatment of diseases.

MSC-EVs have high therapeutic potential for tissue repair. There is evidence of functional engineered EVs isolated from human bone marrow MSCs by introducing lentiviral particles containing BMP2-expressing plasmids. By enhancing the BMP2 signaling cascade in target cells and tissues, the repair of tissues such as myocardium and bone are promoted (115). However, this does not meet all treatment needs. Previous studies on EVs administration have explored the mechanisms of local application (116), systemic administration (117), intrathecal administration (118), vitreous injection (119), and nasal administration (120), but problems such as high demand, low efficiency and strong ectopic effect of EVs have limited its application. Huang et al.'s team encapsulated it in alginate saline gel to promote angiogenesis and regeneration of tissues such as skin, bone, and heart muscle, achieving location or site specificity. These physical wrapping methods resulted in EVs being released within hours of being placed in the desired location (121). Recent studies have used EVs encapsulated in alginate hydrogels for regenerative medicine applications in the treatment of myocardial ischemia or myocardial infarction (122).

## EVs for Individualized Therapy and Diagnosis of Disease

Personalized treatment, also known as personalized medicine or precision medicine, refers to a treatment mode that takes the genetic information and specific disease conditions of patients as guidance and makes targeted treatment plans to improve the cure rate and reduce side effects of patients. Since the “Precision Medicine” plan was put forward in 2015, various countries around the world have launched personalized medicine research projects with huge investment. As a treatment mode of precision medicine, personalized drug screening and drug delivery are required according to patients' physical conditions and disease development. Tailored to the individual situation of each cardiovascular patient, the best treatment plan is designed to achieve a specific medical model that maximizes treatment effect and minimizes adverse reactions (123). Compared with the simple surgical resection for most cardiovascular patients in the past, this treatment method emphasizes patient specific therapies.

Given the limited regenerative capacity of the human heart, stem cell-derived cardiomyocytes are a promising source of alternative cell therapy. iPSC-derived cardiomyocytes (iCMs) have shown potential to attenuate ischemic injury and restore cardiac function in preclinical myocardial infarction models. At present, cell transplantation poses a variety of risks to the recipient. However, the collection and administration of EV's from patient autologous cells could become an effective personalized treatment method.

### Use of Endogenous Circulating EVs in the Diagnosis of Injured Myocardium

The conventional diagnostic methods of heart failure include echocardiography, MRI imaging and identification of myocardial necrosis markers. Exosomes show great diagnostic potential as biomarkers of AMI, and we focus on the diagnostic value of miRNAs and proteins carried by exosomes (124, 125).

miRNAs found in serum and plasma are characterized by stability, time, and tissue specificity. Exosomes, as effective carriers of miRNAs, may provide a new possibility for the diagnosis of AMI. Beg et al. studied the plasma exosome miRNA in patients with heart failure and normal controls with average LVEF of (22.2 ± 7.2) %, and found that the ratio of miR-146a/miR-16 in peripheral blood of patients with heart failure was significantly higher (126). Studies have shown that exosome miR-146a has a cardiomyocyte protective function and has a protective effect on oxidative stress (114). The exosomes isolated from the serum of patients with myocardial infarction contain miRNA-183. Studies have confirmed that the level of miRNA-183 is positively correlated with the degree of myocardial ischemia injury, and classified miRNA-183 as a new biomarker for the diagnosis of myocardial infarction. miRNA-183 is highly enriched in exosomes in patient serum with AMI (127–129). It is worth noting that exosome derived miRNAs can also be detected in urine. In AMI rats, exosome associated miR-1 specifically increased in both blood and urine (91, 130), suggesting that urine derived exosomes may be a new diagnostic method for AMI. The non-invasive extraction of urine may greatly improve the diagnostic rate of myocardial infarction and has a good clinical application prospect. Though miRs is the leading diagnostic marker in the context of exosomes, the potential value of exosomal specific carrier proteins should not be overlooked in the diagnostic evaluation of myocardial infarction.

## Summary and Outlook

EVs contain a variety of proteins, nucleic acids, and lipids, as carriers of intercellular material and information, they can play an important role in influencing the course of disease by regulating interactions between heterogeneous cell microenvironments. EV cargo is effected by disease states, which give circulating EVs the potential as a diagnostic biomarker, which earlier and more accurately reflect the clinical progress of certain diseases, treatment response and prognosis judgement. The study of EVs provides an option for acellular therapy for regeneration and improvement of cardiac function. A large number of experiments have shown that EVs have great therapeutic potential in the treatment of myocardial injury and are also ideal drug delivery tools.

Although many advances have been made in basic research on extracellular vesicle myocardial repair, many challenges remain and some key questions need to be answered in depth before clinical transformation can occur. For example, which cell-derived EVs are most effective at repairing the heart muscle; What are the exact mechanisms of selection and packaging of extracellular vesicle contents; How to regulate the process of extracellular vesicle production; How to efficiently prepare and purify exosomes. How to maximize the retention of EVs in the body so that they can function properly. In addition, allogeneic cells are often used in current studies because of the convenience of obtaining them, but their immunogenicity, individual rejection and *in vivo* safety remain to be confirmed. *In vivo* pharmacokinetic properties, mode of administration, safety, and industrialization must be fully evaluated before clinical transformation can be achieved. The current studies on exosomes as biomarkers in cardiovascular diseases are mostly based on small samples of patients. Moreover, the therapeutic effect of exosomes has only been confirmed in animal experiments and multiple heart-related cells, and no systematic human experiments have been conducted. Despite the above challenges, with the continuous development of cell drug delivery systems, clinical transformation of nano-biomimetic drug delivery systems, the field demonstrates a promising future.

## Author Contributions

CL and NB were responsible for writing the main body of the article. TD and HC retrieved the literature. JT and XL modified the format. DH and YD made charts. PY, JL, ZF, and LW provided ideas and guidance for the writing of the article. All authors contributed to the article and approved the submitted version.

## Funding

This work was supported by the National Natural Science Foundation of China (82104495, 82174161, 81804132, 81803019, and 82002253), Macao Youth Scholars Program (AM2021023), Scientific research projects of Guangdong Bureau of traditional Chinese Medicine (No. 20200513093851), Guangdong Basic and Applied Basic Research Foundation (2021A1515012573 and 2019A1515111108), Science and Technology Foundation of Guangzhou City (202102010257), State Key Laboratory of Dampness Syndrome of Chinese Medicine Research Foundation (SZ2021ZZ21), the TCM Research Fund of Guangdong Provincial Hospital of Chinese Medicine (YN2019MJ15), and the Fund of Science and Technology Innovation Strategy of Guangdong Province (191900105).

## Conflict of Interest

The authors declare that the research was conducted in the absence of any commercial or financial relationships that could be construed as a potential conflict of interest.

## Publisher's Note

All claims expressed in this article are solely those of the authors and do not necessarily represent those of their affiliated organizations, or those of the publisher, the editors and the reviewers. Any product that may be evaluated in this article, or claim that may be made by its manufacturer, is not guaranteed or endorsed by the publisher.
